# Maternal and Child Health handbook and under-6 child overweight in greater Jakarta, Indonesia: a cross-sectional web-based survey

**DOI:** 10.1186/s40795-023-00697-x

**Published:** 2023-03-07

**Authors:** Akiko Saito, Masahide Kondo

**Affiliations:** 1 Department of Nursing, Faculty of Health Science Technology, Bunkyo Gakuin University, 1-19-1 Mukogaoka, Bunkyo, Tokyo, 1138668 Japan; 2 Department of Health Care Policy and Health Economics, University of Tsukuba, 1-1-1 Tennodai, Tsukuba, Ibaraki 3058577 Japan

**Keywords:** Child overweight, Indonesia, Information source, Maternal and Child Health handbook, Child nutrition

## Abstract

**Background:**

In Indonesia, the double burden of child overnutrition and undernutrition is a public health concern. The nationally distributed Maternal and Child Health (MCH) handbook provides child nutrition information to caregivers. We aimed to find mothers’ information sources regarding child nutrition, including the internet and the MCH handbook, and to explore the association between overweight and use of the MCH handbook.

**Method:**

A cross-sectional web-based survey was conducted among mothers with children under 6 years old in Greater Jakarta during 2019. Bivariate and multivariate logistic regression examined the association between child nutrition status and use of the MCH handbook.

**Results:**

Data were collected from 233 children. Overweight, underweight, wasting, and stunting were identified in 36.4%, 22.6%, 26.8%, and 37.6%, respectively. 62.5% of mothers used the MCH handbook, and 88.2% used the internet via a mobile phone. Significantly more cases of overweight were observed among children whose mothers used the MCH handbook (adjusted OR [aOR]: 5.829; 95% Confidential Interval [CI]: 1.618–20.999) whereas no relationship was observed between MCH handbook use and child undernutrition. Significant associations with child overweight were found for mother’s education (tertiary) (aOR: 0.294; 95%CI: 0.098–0.885), employment type (fulltime) (aOR: 0.185; 95%CI: 0.061–0.562), watching television (more than 1 h) (aOR: 4.387; 95%CI: 1.648–11.678) and recognition of child overweight by mother (yes) (aOR: 3.405; 95%CI: 1.05–11.03).

**Conclusion:**

These results indicate the need to support mothers of children exhibiting overnutrition and undernutrition. The MCH handbook should be modified to address this issue.

## Background

Increasing prevalence of child overnutrition is observed in developing countries where undernutrition remains a public health problem [1]. Childhood obesity increases the risk of adulthood cardiovascular disease [2, 3]. Globally, 38 million (5.6%) children under 5 years of age were estimated to be overweight or obese in 2019 [1]. Indonesia’s 2018 National Basic Health Research Survey (RISKESDAS) reported that 8.0% of children under 5 years of age were overweight, while stunting, wasting, and underweight were identified in 29.8%, 10.3%, and 17.7%, respectively [4].

To tackle the double burden of overnutrition and undernutrition, it is crucial to deliver the necessary information to mothers or caregivers. However, little is known about the sources of information about child nutrition used by caregivers in Indonesia. In rural Indonesia, health facilities are major sources of information about stunting for mothers [5]. Several previous studies have investigated sources of information for pregnant women regarding nutrition, [6–8] and multiple sources have been identified, including health professionals and the internet [6].

Internet technology is widely used in Indonesia, and 71% of people were found to possess smartphones in urban areas in 2018 [9]. Consequently, it may be expected that many mothers seek information about child nutrition from the internet. Some previous studies have reported that the internet is used as an information source by pregnant women in Indonesia [6–8] and a qualitative study reported that 17 of 23 pregnant women sought nutrition information through the internet [6].

In addition, the nationally distributed Maternal and Child Health (MCH) handbook is also a major source of information about child nutrition in Indonesia. The MCH handbook is a home-based printed booklet that is distributed to all pregnant women at their first antenatal care visit. This handbook functions as a maternal and child health record and information book, and contains nutrition information about various topics, such as exclusive breastfeeding, complementary food, and healthy diets. However, the extent to which mothers use the handbook as a source of information is currently unclear.

Previous studies have investigated the risk factors for child overweight and obesity, [11–17, 17–19] and excess energy intake and low physical activity level have been identified in a number of studies. [10, 12, 17, 19]. The reported associations between parents’ education level, [11, 13, 17, 19] economic level [14, 17, 20] and child obesity have been inconsistent among previous studies. In previous studies in Indonesia, being a male child, having parents who are overweight, having a father with a university education, [13, 14] being part of a family in the higher economic quintile, [14, 17] being stunted, [18, 20] being an urban resident, [13, 16, 17] having a low physical activity level, [17] consuming ultra-processed food, [13] and frequent intake of fried foods [19] have been identified as risk factors for child obesity. However, the way in which the MCH handbook relates to child nutrition in current conditions regarding the increasing prevalence of child overnutrition has not been investigated. Osaki et al. (2019) reported that the prevalence of stunting and underweight in children were significantly lower in families that were guided and sensitized using the MCH handbook compared with families that were not guided or sensitized [10]. However, it is also necessary to explore the association between the MCH handbook and child overweight.

Thus, in the current study, we sought to clarify the sources of information about child nutrition used by mothers, including the internet and the MCH handbook, and to explore the association between overweight and use of the MCH handbook. Although the main focus of the current study was overweight, undernutrition was also included in the analysis.

## Methods

A cross-sectional survey was conducted among mothers of children under 6 years old in Greater Jakarta from 7 to 10 May 2019.

Mothers who were 16 years old or older and living in Greater Jakarta were recruited through a local web survey agency. No exclusion criteria were set as long as participants meet all inclusion criteria. The web-based survey was purposefully chosen to examine an association between overweight and the MCH handbook as an information source among mothers who have access to internet. Because smartphone possession rate is higher [9] and more child overweight exists in urban than rural area, [13, 16, 17] Greater Jakarta was selected as study site. It is known that child overweight in Indonesia is more prevalent in families containing mothers with higher economic level, [14, 17, 20] and a higher level of education, [11, 13, 17, 19] and the participants in the web-based survey were expected to have these characteristics. The target number of participants, 180 mothers, was determined according to sample size calculation and budgetary consideration. An invitation was sent to all mobile panels (30,851 eligible panels out of 963,197 panels as of 2018) who registered to the web survey agency. The first page of the survey contained information, describing the study and asking for their voluntary participation. All participants provided informed consent by reading and responding. Ethics Committee, Faculty of Health Science Technology & Graduate School of Health Care Science, Bunkyo Gakuin University permitted this research (#2018-0034).

In a structured questionnaire, mothers were asked to provide the following information: mother’s sociodemographic information, child’s age, sex, weight (kg), height (cm), hours of watching television, ownership of the MCH handbook, and nutrition practice. Mother’s sociodemographic data included mother’s age, education level, employment type, and household monthly income. Sources of information about child nutrition were collected through multiple choice answers regarding use of the MCH handbook, internet via mobile phone, internet via computer, books or magazines, family members, friends, health professionals, and other sources. Sources of anthropometric data were not identified in the questionnaire.

The World Health Organization 2006 Growth Standard was applied to classify child nutrition status. Child overweight was defined as a weight for height z-score > 2 standard deviations (SD), child stunting was defined as a height/length for age z-score < − 2 SD, child wasting was defined as a weight for height z-score < 2 SD, and child underweight was defined as a weight for age z-score < − 2 SD [22].

Descriptive analysis was performed to present the prevalence of overweight, stunting, underweight, wasting, and normal (not malnourished) as well as the distribution of each variable. Because the analysis was based on the child, a mother’s data were used twice if they provided information about more than one child. Sources of information for child nutrition were also described in percentages (%). The Odds Ratio [OR] and 95% Confidential Interval [CI] for the association of factors related to overweight and each nutrition status were estimated using bivariate and multivariate logistic regression analysis. Appropriate cut-off values were applied to create binary variables for all items. The Statistical Package for Social Science (SPSS) software version 28.0 (IBM, Armonk, NY, USA) as used to perform statistical analysis.

## Results

Data were collected for a total of 233 children from 180 mothers. The distributions of overweight, stunting, underweight, and wasting are presented in Table 1. The high prevalence observed for child overweight (36.4%) was expected, because the study was designed to capture a population at increased risk of child overweight. However, surprisingly, stunting, underweight, and wasting also had relatively high prevalence rates of 22.6%, 26.8%, and 37.6%, respectively.

**Table 1:** Distribution of overweight, stunting, underweight, and wasting (N = 233)

	n	%
Overweight		
Yes	80	36.4
No	140	63.6
Missing	13	
Stunting		
Yes	85	37.6
No	141	62.4
Missing	7	
Underweight		
Yes	51	22.6
No	175	77.4
Missing	7	
Wasting		
Yes	37	16.8
No	183	83.2
Missing	13	
Normal		
Yes	73	34.8
No	137	65.2
Missing	13	

A description of child sociodemographic characteristics and nutrition practice by nutrition status is provided in Table 2. Nearly half of the children were 0–2 years old and the other half were 3 years old or older. 65.2% of children had mothers that were aged 30 years and older, and 34.8% of children had mothers aged 20–29 years old. None of the mothers were teenagers. Because it has previously been reported that a higher prevalence of child overweight is found in families with mothers who have higher levels of education [11, 13, 17, 19] and economic level [14, 17, 20] in Indonesia, we sought to identify these mothers in the current study. Therefore, most households had a relatively high monthly income, and most mothers had a high level of education. Of the households, 41.6% had a monthly income of Rp. 1.250.001-Rp. 4.000.000, and 58.4% had a monthly income of Rp.4,000,001 or more. All mothers had completed secondary education and 76.8% of them had completed tertiary and higher education. 87.4% of mothers possessed the MCH handbook. 51.8% of the children watched television for less than 1 h a day, and 48.2% watched television for 1 h or more a day.

**Table 2:** Sociodemographic characteristics and nutrition practice of children and mothers by nutrition status

	Overall	Overweight	Stunting	Underweight	Wasting	Normal
**Child’s age**						
0–2 years old	110 (47.2)	39 (48.8)	29 (34.1)	16 (31.4)	16 (43.2)	42 (57.5)
3 years and over	123 (52.8)	41 (51.2)	56 (65.9)	35 (68.6)	21 (56.8)	31 (42.5)
**Child's sex**						
Boy	114 (48.9)	32 (40)	47 (55.3)	30 (58.8)	20 (54.1)	36 (49.3)
Girl	119 (51.1)	48 (60)	38 (44.7)	21 (41.2)	17 (45.9)	37 (50.7)
**Mother’s age**						
20–29 years old	82 (35.2)	29 (36.3)	31 (36.5)	24 (47.1)	15 (40.5)	22 (30.1)
30 years old and over	151 (64.8)	51 (63.7)	54 (63.5)	27 (52.9)	22 (59.5)	51 (69.9)
**Mother’s marital status**						
Married	229 (98.3)	79 (98.8)	85 (100)	51 (100)	37 (100)	71 (97.3)
Not married or widowed	4 (1.7)	1 (1.3)	0 (0)	0 (0)	0 (0)	2 (2.7)
**Mother’s education level**						
Completed secondary school	54 (23.2)	21 (26.3)	19 (22.4)	11 (21.6)	13 (35.1)	9 (12.3)
Completed tertiary or higher education	179 (76.8)	59 (73.8)	66 (77.6)	40 (78.4)	24 (64.9)	64 (87.7)
**Mother’s employed type**						
Not fulltime or housewife	145 (62.2)	54 (67.5)	50 (58.8)	29 (56.9)	15 (40.5)	49 (67.1)
Fulltime worker	88 (37.8)	26 (32.5)	35 (41.2)	22 (43.1)	22 (59.5)	24 (32.9)
**Monthly household income**						
Rp. 1.250.001-Rp. 4.000.000	97 (41.6)	33 (41.3)	33 (38.8)	22 (43.1)	19 (51.4)	28 (38.4)
Rp. 4.000.001 or more	136 (58.4)	47 (58.8)	52 (61.2)	29 (56.9)	18 (48.6)	45 (61.6)
**Owns MCHHB**						
No	30 (13.2)	11 (13.9)	9 (10.6)	5 (9.8)	6 (16.2)	7 (10.1)
Yes	198 (86.8)	68 (86.1)	76 (89.4)	46 (90.2)	31 (83.8)	62 (89.9)
**Hours of television watching a day**						
Less than 1 hour	117 (50.2)	33 (41.3)	47 (55.3)	35 (68.6)	22 (59.5)	36 (49.3)
1 hour or more	116 (49.8)	47 (58.8)	38 (44.7)	16 (31.4)	15 (40.5)	37 (50.7)
**Told child is overweight by medical worker**						
No	188 (87.0)	51 (75)	62 (78.5)	45 (91.8)	34 (94.4)	68 (97.1)
Yes	28 (13.04)	17 (25)	17 (21.5)	4 (8.2)	2 (5.6)	2 (2.9)
**Breastfeeding before 6 months old**						
No	23 (9.9)	8 (10.1)	4 (4.8)	2 (3.9)	4 (10.8)	9 (12.3)
Yes	209 (90.1)	71 (89.9)	80 (95.2)	49 (96.1)	33 (89.2)	64 (87.7)
**Formula milk before 6 months old**						
No	123 (53.0)	44 (55.7)	46 (54.8)	33 (64.7)	21 (56.8)	32 (43.8)
Yes	109 (47.0)	35 (44.3)	38 (45.2)	18 (35.3)	16 (43.2)	41 (56.2)
**Water before 6 months old**						
No	210 (90.5)	69 (87.3)	72 (85.7)	49 (96.1)	36 (97.3)	66 (90.4)
Yes	22 (9.5)	10 (12.7)	12 (14.3)	2 (3.9)	1 (2.7)	7 (9.6)
**Sweetened beverage before 6 months old**						
No	209 (90.1)	69 (87.3)	73 (86.9)	50 (98)	35 (94.6)	66 (90.4)
Yes	23 (9.9)	10 (12.7)	11 (13.1)	1 (2)	2 (5.4)	7 (9.6)
**Complementary food before 6 months old**						
No	178 (76.7)	57 (72.2)	64 (76.2)	46 (90.2)	35 (94.6)	54 (74)
Yes	54 (23.3)	22 (27.8)	20 (23.8)	5 (9.8)	2 (5.4)	19 (26)
**Junk food**						
No	216 (93.1)	70 (88.6)	75 (89.3)	51 (100)	37 (100)	69 (94.5)
Yes	16 (6.9)	9 (11.4)	9 (10.7)	0 (0)	0 (0)	4 (5.5)
**Snacks**†						
Never	27 (12.5)	12 (16.2)	14 (16.9)	11 (22.0)	5 (13.5)	5 (7.8)
Once a day or more	189 (87.5)	62 (83.8)	69 (83.1)	39 (78.0)	32 (86.5)	59 (92.2)
**Eat outside**‡						
Less than few times a week	111 (51.4)	40 (54.1)	48 (57.8)	36 (72.0)	19 (51.4)	26 (40.6)
Few times a week or more	105 (48.6)	34 (45.9)	35 (42.2)	14 (28.0)	18 (48.6)	38 (59.4)
**Takeaway food**‡						
Less than few times a week	115 (53.2)	44 (59.5)	47 (56.6)	33 (66.0)	19 (51.4)	29 (45.3)
Few times a week or more	101 (46.8)	30 (40.5)	36 (43.4)	17 (34.0)	18 (48.6)	35 (54.7)
Stunting						
No		41 (52.6)				
Yes		37 (47.4)				

MCH handbook: Maternal and Child Health handbook

† 6 months and older children only

‡ 1 year and older children only

Table 3 shows the sources of information about child nutrition by nutrition status. 65.2% of mothers used the MCH handbook. As expected, accessing the internet via mobile phone was the most prevalent information source of information, used by 88.2% of mothers. Of mothers with an overweight child, 86.6% and 65.1% sought information from the MCH handbook and the internet via mobile phone, respectively.

**Table 3:** Information resource of child nutrition by nutrition status (N = 233)

	Overall	Overweight	Stunting	Underweight	Wasting	Normal		
	n (%)	n (%)	n (%)	n (%)	n (%)	n (%)		
Information from MCH handbook								
No	81(34.8)	22 (27.5)	23 (27.1)	17 (33.3)	13 (35.1)	29 (39.7)		
Yes	152 (65.2)	58 (72.5)	62 (72.9)	34 (66.7)	24 (64.9)	44 (60.3)		
Information from internet via mobile phone								
No	26 (11.2)	9 (11.25)	12 (14.1)	7 (13.7)	2 (5.4)	8 (11.0)		
Yes	207 (88.8)	71 (88.8)	73 (85.9)	44 (86.3)	35 (94.6)	65 (89.0)		
Information from internet via computer						0		
No	117 (50.2)	38 (47.5)	43 (50.6)	29 (56.9)	24 (64.9)	33 (45.2)		
Yes	116 (49.8)	42 (52.5)	42 (49.4)	22 (43.1)	13 (35.1)	40 (54.8)		
Information from books/magazines								
No	109 (46.8)	40 (50)	44 (51.8)	25 (49.0)	20 (54.1)	33 (45.2)		
Yes	124 (53.2)	40 (50)	41 (48.2)	26 (51.0)	17 (45.9)	40 (54.8)		
Information from family members								
No	79 (33.9)	30 (37.5)	24 (28.2)	14 (27.5)	12 (32.4)	26 (35.6)		
Yes	154 (66.1)	50 (62.5)	61 (71.8)	37 (72.5)	25 (67.6)	47 (64.4)		
Information from friends								
No	110 (47.2)	38 (47.5)	37 (43.5)	22 (43.1)	16 (43.2)	37 (50.7)		
Yes	123 (52.8)	42 (52.5)	48 (56.5)	29 (56.9)	21 (56.8)	36 (49.3)		
Information from medical workers								
No	117 (50.2)	45 (56.25)	44 (51.8)	20 (39.2)	19 (51.4)	36 (49.3)		
Yes	116 (49.8)	35 (43.8)	41 (48.2)	31 (60.8)	18 (48.6)	37 (50.7)		
Information from others								
No	230 (98.7)	79 (98.75)	84 (98.8)	50 (98.0)	36 (97.3)	72 (98.6)		
Yes	3 (1.3)	1 (1.25)	1 (1.2)	1 (2.0)	1 (2.7)	1 (1.4)		

MCH handbook: Maternal and Child Health handbook

The results of bivariate and multivariate analysis are presented in Tables 4 and 5, respectively. Multivariate analysis revealed that there was a significantly higher prevalence of overweight children among mothers who used the MCH handbook as an information source (adjusted OR [aOR]: 5.829, 95%CI; 1.618–20.999). However, no significant relationship was observed between the MCH handbook and child underweight in the current study. A significant association with child overweight was seen with mother’s education (tertiary) (aOR; 0.294, 95%CI; 0.098–0.885), employment type (fulltime) (aOR; 0.185, 95%CI; 0.061–0.562) and watching television (more than 1 h) (aOR; 4.387, 95%CI; 1.648–11.678). In addition, the results revealed that mother’s recognition of their child’s nutrition status accurately reflected their child’s nutrition status; thus, when mothers thought that their child was overweight, the child tended to be overweight (aOR; 3.405, 95%CI; 1.05–11.03). Child’s age (3 years and older) was significantly associated with stunting (aOR; 6.211, 95%CI; 2.69–14.34) and underweight (aOR; 5.129, 95%CI; 2.012–13.401). Mother’s age (30 years and older) was associated with underweight (aOR; 0.318, 95%CI; 0.136–0.741). Complementary food given before 6 months old was associated with wasting (aOR; 0.157, 95%CI; 0.029–0.852). In the current study, no association was observed between use of the MCH handbook and undernutrition.

**Table 4:** Results of bivariate logistic regression analysis of nutrition status

	Overweight			Stunting			Underweight			Wasting		
	Odds	95%CI		Odds	95%CI		Odds	95%CI		Odds	95%CI	
Child sex (girl)	1.731	0.991	3.021	0.691	0.403	1.188	0.617	0.328	1.161	0.77	0.379	1.565
Child age (3 years or older)	0.911	0.526	1.579	2.607	1.491	4.559	2.539	1.31	4.921	1.216	0.596	2.478
Mother's age (30 years or older)	0.977	0.551	1.731	0.871	0.496	.1.53	0.502	0.266	0.949	0.789	0.383	1.626
Mother’s education (Tertiary)	0.734	0.385	1.398	0.979	0.512	1.87	1.043	0.489	2.222	0.468	0.218	1.006
Mother’s employment (Fulltime)	0.661	0.372	1.176	1.536	0.779	2.362	1.418	0.751	2.676	2.794	1.355	5.76
Monthly household income (Rp. 4,000,001 or more)	1.068	0.612	1.864	1.134	0.654	1.965	0.879	0.467	1.652	0.643	0.317	1.307
Television (1 hour or more)	1.955	1.12	3.414	0.691	0.403	1.188	0.359	0.185	0.697	0.689	0.336	1.412
Owns MCH handbook (yes)	0.824	0.362	1.878	1.456	0.63	3.366	1.512	0.546	4.189	0.691	0.258	1.852
Information from MCH handbook (yes)	1.558	0.856	2.835	1.724	0.959	3.098	1.07	0.553	2.071	0.923	0.44	1.938
Information from internet via mobile phone (yes)	1.09	0.462	2.574	0.618	0.268	1.425	0.721	0.283	1.835	2.642	0.596	11.699
Information from internet via computer (yes)	1.275	0.736	2.211	0.936	0.546	1.604	0.684	0.365	1.283	0.513	0.246	1.069
Information from books/magazines (yes)	0.867	0.5	1.502	0.711	0.414	1.22	0.876	0.469	1.635	0.737	0.363	1.498
Information from family members (yes)	0.842	0.475	1.493	1.485	0.829	2.661	1.45	0.728	2.887	1.148	0.541	2.434
Information from friends (yes)	1.044	0.602	1.809	1.175	0.684	2.018	1.162	0.62	2.179	1.27	0.623	2.589
Information from medical workers (yes)	0.674	0.388	1.171	0.893	0.521	1.53	1.758	0.931	3.32	0.937	0.462	1.9
Information from others (yes)	0.873	0.078	9.786	0.827	0.074	9.265	1.73	0.154	19.475	2.514	0.222	28.468
Breastfed before 6 months old (yes)	0.908	0.359	2.296	3.115	1.022	9.494	3.363	0.761	14.856	0.85	0.269	2.689
Formula milk given before 6 months old (yes)	0.972	0.558	1.693	0.887	0.516	1.525	0.533	0.279	1.018	0.929	0.455	1.895
Water given before 6 months old (yes)	2.11	0.819	5.436	2.183	0.899	5.301	0.314	0.071	1.393	0.253	0.033	1.958
Sweetened beverage given before 6 months old (yes)	2.11	0.819	5.436	1.62	0.681	3.855	0.138	0.018	1.051	0.555	0.123	2.51
Complementary food given before 6 months old (yes)	1.775	0.922	3.418	1.064	0.562	2.015	0.294	0.11	0.784	0.174	0.04	0.752
Junk food given before 6 months old (yes)	4.371	1.3	14.697	2.297	0.822	6.418						
Snacks given once a day or more (yes) †	0.674	0.297	1.529	0.379	0.156	0.922	0.289	0.119	0.706	0.971	0.342	2.758
Eat outside few times a week or more (yes) ‡	0.877	0.495	1.553	0.602	0.344	1.054	0.298	0.149	0.596	1.03	0.505	2.101
Eat take away food few times a week or more (yes) ‡	0.682	0.383	1.215	0.766	0.439	1.337	0.484	0.249	0.938	1.107	0.543	2.259
Mother thinks child is overweight	7.167	2.675	19.202									
Stunting	1.973	1.113	3.497									
Overweight				1.973	1.113	3.497						

MCH Handbook; Maternal and Child Health Handbook

† 6 months and older children only

‡ 1 year and older children only

**Table 5:** Results of multivariate logistic regression analysis of nutrition status

	Overweight			Stunting			Underweight			Wasting		
	Odds	95%CI		Odds	95%CI		Odds	95%CI		Odds	95%CI	
Child sex (girl)	1.432	0.642	3.197	0.654	0.322	1.327	0.728	0.332	1.6	0.676	0.3	1.523
Child age (3 years or older)	0.509	0.208	1.242	6.211	2.69	14.34	5.192	2.012	13.401	0.989	0.409	2.392
Mother's age (30 years or older)	0.974	0.419	2.264	0.556	0.262	1.178	0.318	0.136	0.741	0.964	0.412	2.257
Mother’s education (Tertiary)	0.294	0.098	0.885	2.093	0.789	5.554	2.55	0.884	7.356	0.655	0.236	1.819
Mother’s employment (Fulltime)	0.185	0.061	0.562	1.687	0.682	4.169	1.213	0.439	3.351	2.215	0.812	6.046
Monthly household income (Rp. 4,000,001 or more)	1.207	0.459	3.168	0.796	0.357	1.777	0.702	0.295	1.671	0.758	0.31	1.851
Television (1 hour or more)	4.387	1.648	11.678	0.493	0.201	1.207	0.504	0.185	1.369	0.782	0.287	2.127
Owns MCH handbook (yes)	0.469	0.1	2.199	1.143	0.276	4.727	2.395	0.467	12.28	0.609	0.149	2.487
Information from MCH handbook (yes)	4.133	1.252	13.639	1.555	0.598	4.043	0.886	0.314	2.501	1.021	0.327	3.192
Information from internet via mobile phone (yes)	2.179	0.589	8.068	0.46	0.146	1.448	0.718	0.192	2.678	4.068	0.735	22.507
Information from internet via computer (yes)	1.963	0.762	5.058	0.927	0.4	2.148	0.657	0.259	1.661	0.51	0.187	1.39
Information from books/magazines (yes)	0.522	0.173	1.57	0.564	0.217	1.464	0.586	0.2	1.718	0.768	0.272	2.167
Information from family members (yes)	0.425	0.131	1.376	1.749	0.648	4.723	1.669	0.519	5.364	1.296	0.387	4.342
Information from friends (yes)	2.192	0.759	6.334	1.215	0.523	2.823	1.796	0.661	4.876	1.277	0.437	3.729
Information from medical workers (yes)	0.483	0.183	1.277	1.387	0.599	3.211	2.326	0.887	6.101	1.225	0.46	3.257
Information from others (yes)	2.697	0.096	75.984	0.863	0.039	19.282	2.144	0.102	44.839	11.238	0.423	298.68
Breastfed before 6 months old (yes)	2.323	0.447	12.08	2.92	0.634	13.442	0.984	0.165	5.875	0.553	0.124	2.467
Formula milk given before 6 months old (yes)	0.498	0.184	1.348	0.915	0.402	2.081	0.774	0.312	1.918	1.283	0.512	3.212
Water given before 6 months old (yes)	1.878	0.247	14.259	3.595	0.606	21.304	1.908	0.115	31.751	1.308	0.092	18.539
Sweetened beverage given before 6 months old (yes)	0.204	0.019	2.236	0.641	0.109	3.765	0.282	0.019	4.188	0.752	0.101	5.623
Complementary food given before 6 months old (yes)	2.467	0.827	7.358	0.711	0.268	1.881	0.485	0.146	1.616	0.157	0.029	0.852
Junk food given before 6 months old (yes)	8.154	0.529	125.81	3.419	0.506	23.084						
Snacks given once a day or more (yes) †	0.429	0.125	1.474	0.374	0.108	1.298	0.297	0.082	1.076	1.555	0.44	5.499
Eat outside few times a week or more (yes) ‡	0.581	0.193	1.753	0.552	0.208	1.468	0.322	0.1	1.031	1.3	0.425	3.976
Eat take away food few times a week or more (yes) ‡	0.47	0.158	1.393	0.955	0.363	2.508	1.487	0.498	4.442	1.204	0.42	3.454
Mother thinks child is overweight	3.405	1.05	11.038									
Stunting	2.184	0.914	5.218									
Overweight				3.203	1.436	7.141						

MCH handbook; Maternal and Child Health handbook

† 6 months and older children only

‡ 1 year and older children only

## Discussion

The current study revealed that 65.2% of mothers used the MCH handbook as a source of information about child nutrition, whereas 88.2% of mothers used the internet via mobile phone as a source of information about child nutrition. In addition, the results revealed that children whose mothers used the MCH handbook as an information source were more likely to be overweight than children of mothers who did not use the MCH handbook.

The observed prevalence (36.4%) of child overweight in the current study was higher than that reported in previous studies [4, 18, 22, 23]. A report published by the UNICEF, the World Health Organization, and the World Bank Group estimated the prevalence of overweight/obesity in children under 5 years old as 11.1% in Indonesia [3]. We sought to target children whose mothers had a high level of education and high economic level to provide an adequate sample of overweight children. Therefore, a high prevalence of child overweight was anticipated. However, surprisingly high prevalence rates of stunting (37.6%), underweight (22.6%) and wasting (16.8%) were also observed. These results indicate the importance of dealing with both overnutrition and undernutrition of children.

The current study revealed that 65.2% of mothers sought information about child nutrition from the MCH handbook, whereas 88.2% of mothers sought information from the internet via mobile phone. We aimed to identify the extent to which mothers use the MCH handbook in the era of internet technology, and expected that the internet would be the most frequently used information source. The current results indicate that mothers still value the MCH handbook as a source of information, although the internet is becoming a popular source. However, while the internet enables mothers to access information in a convenient way, it can be difficult to find reliable information. In contrast, the national MCH handbook is a reliable source of accurate information. The current findings are encouraging for policy makers and health professionals, indicating that the MCH handbook is an effective tool for delivering important information to mothers in Indonesia.

The current study revealed a significant association between child overweight and mothers use of the MCH handbook as a source of information about child nutrition (aOR; 4.133, 95%CI; 1.252–13.639) whereas no association was found between undernutrition and the use of the MCH handbook. To the best of our knowledge, this is the first study to investigate the relationship between child overweight and use of the MCH handbook as a source of information in Indonesia. This finding suggests the importance of examining the contents of the MCH handbook. The Indonesian MCH handbook provides information about child nutrition and recommends a healthy diet. However, information regarding the prevention of child overweight/obesity is not clearly described. The handbook was officially approved as a national home-based record in 2004 [25], when undernutrition was the main concern and revisions were made afterwards. Indonesia, like many other developing countries, has experienced a transition in nutritional status because of the introduction of western food and corresponding lifestyle changes. Given this new context, concise and accurate content regarding the prevention of child overweight and obesity should be added to the MCH handbook.

To deliver the necessary information to mothers, it may be useful to create links between the printed MCH handbook and internet websites. Furthermore, an MCH handbook smartphone app could be made available to mothers. Positive outcomes of internet technology use for information delivery in the field of health in Indonesia have previously been reported, including improved women’s knowledge and behavior regarding hygiene [25].

The internet and the MCH handbook each have advantages and disadvantages as sources of information. The MCH handbook is a national service and is distributed free of charge, whereas internet access entails a cost for mothers, including the cost of a device, and wifi/internet access may not be always stable. Regarding information capacity, space is limited in the MCH handbook, whereas the internet has a vast information capacity. Therefore, essential information may be better presented in the printed MCH handbook or via a smartphone app. Further information could then be provided via the internet.

### Study strength and limitations

This study updated existing knowledge regarding mother’s information source of child nutrition. It designed to reflect the current situation of mothers who uses internet daily. Furthermore, studies on the MCH handbook scarce all over the world. The current study adds new findings about the MCH handbook. Then it handled both overnutrition and undernutrition. The double burden of overnutrition and undernutrition is the realistic situation of Indonesia [4] as well as other developing countries [1]. Both at nation level and individual level, coexistence of undernutrition and overnutrition has been recognized [1, 4, 19, 25, 25]. The study adds newer knowledge to tackle such situation.

The current study involved several limitations. First, the study sample was not representative of children in Indonesia, or any other general population, limiting the generalizability of the results. Due to web-based survey, the study sample is limited to internet users that is biased to high education background and monthly household income. Consequently, mothers who uses internet but are not high education background or monthly household income are excluded from this study. However, this was a trade-off with our intended participants that enabled us to analyze relationship between child overweight and the MCH handbook use as information source of child nutrition in the era of internet technology. Second, some important or potentially confounding variables such as father’s education level and parents’ nutritional status were not collected due to budgetary restriction. Third, recall bias might have contributed to the high prevalence observed. Anthropometric data were entered by mothers regardless of the data source. A time lag might have occurred between the age of child at the time of anthropometric measurement and the actual age of the child at the time the survey was completed.

## Conclusion

In the current study, we attempted to determine the prevalence of use of the MCH handbook as a source of information about child nutrition in the era of internet technology, and its relationship with child overweight. The results revealed that many mothers use the MCH handbook despite having access to the internet. However, the current results revealed that use of the MCH handbook was positively associated with child overweight. The content of the handbook should be examined in more depth to determine whether it contains unsuitable information regarding the double burden of child overweight and undernutrition identified in this study. In addition, it would be meaningful to discuss how to deliver information regarding child overweight to mothers and caregivers. Covering related topics in the national MCH handbook and creating a linkage between the MCH handbook and the internet may be useful. Future studies with a larger sample size may be necessary for assessing the situation more accurately.

## Data Availability

The datasets used and/or analysed during the current study are available from the corresponding author on reasonable request.

## List of abbreviations

aOR: adjusted Odds Ratio
CI: Confidential Interval
MCH: Maternal and Child Health
OR: Odds Ratio

## References

[1] UNICEF, World Health Organization, The World Bank Group (2020). Levels and trends in child malnutrition: key findings of the 2021 edition of the joint child malnutrition estimates.

[2] Umer A, Kelley GA, Cottrell LE, Giacobbi P, Innes KE, Lilly CL (2017). Childhood obesity and adult cardiovascular disease risk factors: a systematic review with meta-analysis. BMC Public Health.

[3] Sommer A, Twig G (2018). The impact of childhood and adolescent obesity on cardiovascular risk in adulthood: a systematic review. Curr Diab Rep.

[4] Badan Penelitian dan Pengembangan Kesehatan (2019). Laporan Nasional Riskesdas 2018/ Badan Penelitian dan Pengembangan Kesehatan.

[5] West J, Syafiq A, Crookston B (2018). Stunting-related knowledge: exploring sources of and factors associated with accessing stunting-related knowledge among mothers in rural Indonesia. Health.

[6] Rahmawati W, Willcox JC, van der Pligt P, Worsley A (2021). Nutrition information-seeking behaviour of indonesian pregnant women. Midwifery.

[7] Rahmawati W, van der Pligt P, Willcox JC, Worsley AF (2021). Sources of nutrition information for indonesian women during pregnancy: how is information sought and provided?. Public Health Nutr.

[8] Wijaya-Erhardt M, Muslimatun S, Erhardt JG (2014). Effect of an educational intervention related to health and nutrition on pregnant women in the villages of Central Java Province, Indonesia. Health Educ J.

[9] Asosiasi Penyelenggara Jasa Internet Indonesa (APJII) (2017). Penetrasi dan Perilaku Pengguna Internet Indonesia: Survei 2017 (Survei 2017).

[10] Osaki K, Hattori T, Toda A, Mulati E, Hermawan L, Pritasari K et al. Maternal and Child Health Handbook use for maternal and child care: a cluster randomized controlled study in rural Java, Indonesia. J Public Health (Oxf). 2019 Mar 1;41(1):170–182. 10.1093/pubmed/fdx175 10.1093/pubmed/fdx175 PMC6459363 29325171

[11] Woo Baidal JA, Locks LM, Cheng ER, Blake-Lamb TL, Perkins ME, Taveras EM (2016). Risk factors for childhood obesity in the first 1,000 days: a systematic review. Am J Prev Med.

[12] Brown T, Moore TH, Hooper L, Gao Y, Zayegh A, Ijaz S et al. Interventions for preventing obesity in children. Cochrane Database Syst Rev. 2019 Jul 23;7(7):CD001871. 10.1002/14651858.CD001871.pub4 10.1002/14651858.CD001871.pub4 PMC6646867 31332776

[13] Oddo VM, Maehara M, Rah JH (2019). Overweight in Indonesia: an observational study of trends and risk factors among adults and children. BMJ Open.

[14] Rachmi CN, Li M, Baur LA (2017). Overweight and obesity in Indonesia: prevalence and risk factors—a literature review. Public Health.

[15] Patro-Gołąb B, Zalewski BM, Kołodziej M, Kouwenhoven S, Poston L, Godfrey KM, et al. Nutritional interventions or exposures in infants and children aged up to 3 years and their effects on subsequent risk of overweight, obesity and body fat: a systematic review of systematic reviews. Obes Rev. 2016 Dec;17(12):1245–57. 10.1111/obr.12476 10.1111/obr.12476 PMC5325317 27749991

[16] Sandjaja S, Budiman B, Harahap H, Ernawati F, Soekatri M, Widodo Y, Harahap H, Sandjaja S, Soekatri M, Khouw I, Deurenberg P et al. Association of energy intake and physical activity with overweight among Indonesian children 6–12 years of age. *Asia Pac J Clin Nutr*. 2018;27(1):211-6. 10.6133/apjcn.032017.05 10.6133/apjcn.032017.05 29222901

[17] Aitsi-Selmi A, Bell R, Shipley MJ, Marmot MG (2014). Education modifies the association of wealth with obesity in women in middle-income but not low-income countries: an interaction study using seven national datasets, 2005–2010. PLoS ONE.

[18] Rachmi CN, Agho KE, Li M, Baur LA (2016). Stunting, underweight and overweight in children aged 2.0–4.9 years in Indonesia: prevalence trends and associated risk factors. PLoS ONE.

[19] World Health Organization. WHO child growth standards: length/height-for-age, weight-for-age, weight-for-length, weight-for-height and body mass index-for-age: methods and development. Geneva: World Health Organization. ; 2006. https://www.who.int/publications/i/item/924154693X. Accessed 12 September 2022.

[20] Ayuningtyas D, Hapsari D, Rachmalina R, Amir V, Rachmawati R, Kusuma D (2022). Geographic and socioeconomic disparity in child undernutrition across 514 districts in Indonesia. Nutrients.

[21] Mahmudiono T, Nindya TS, Andrias DR, Megatsari H, Rosenkranz RR (2018). Household food insecurity as a predictor of stunted children and overweight/obese mothers (SCOWT) in urban Indonesia. Nutrients.

[22] Niedfeldt HJ, Beckstead E, Chahalis E, Jensen M, Reher B, Torres S et al. Use of Technology to Access Health Information/Services and Subsequent Association With WASH (Water Access, Sanitation, and Hygiene) Knowledge and Behaviors Among Women With Children Under 2 Years of Age in Indonesia: Cross-sectional Study. JMIR Public Health Surveill. 2021 Jan 14;7(1):e19349.10.2196/19349 10.2196/19349 PMC7843201 33443485

[23] Japan International Cooperation Agency. Technical brief global promotion of maternal and child health handbook issue 2, INDONESIA roles of MCH handbook in service uptake based on Indonesia National Health Survey. Tokyo. Japan International Cooperation Agency. 2016. https://libopac.jica.go.jp/images/report/1000030133_02.pdf. Accessed 14 July 14 2022.

[24] Farah AM, Nour TY, Endris BS, Gebreyesus SH. Concurrence of stunting and overweight/obesity among children: Evidence from Ethiopia. PLoS One. 2021 Jan 15;16(1):e0245456. 10.1371/journal.pone.0245456 10.1371/journal.pone.0245456 PMC7810347 33449970

[25] Zhang N, Bécares L, Chandola T. Patterns and Determinants of Double-Burden of Malnutrition among Rural Children: Evidence from China. PLoS One. 2016 Jul 8;11(7):e0158119. 10.1371/journal.pone.0158119 10.1371/journal.pone.0158119 PMC4938417 27391448

